# A Case Report of Valacyclovir-Associated Neurotoxicity

**DOI:** 10.3390/microorganisms14040755

**Published:** 2026-03-27

**Authors:** Kim Hoang, Tatiana Barrera, Miki Watanabe, Herman Joseph Johannesmeyer

**Affiliations:** 1 Irvine Medical Center, University of California, Orange, CA 92868, USA; 2 School of Medicine, University of California, Irvine, CA 92617, USA; 3 School of Pharmacy and Pharmaceutical Sciences, University of California, Irvine, CA 92617, USA

**Keywords:** valacyclovir, neurotoxicity, encephalopathy, overdose, dialysis

## Abstract

We report the case of a 69-year-old Hispanic female with end-stage renal disease (ESRD) on hemodialysis and a complex medical history who presented with acute altered mental status shortly after initiating valacyclovir at a non-renally adjusted dose. The patient was admitted to inpatient services and treated with a presumptive diagnosis of valacyclovir-associated neurotoxicity (VAN). The patient received hemodialysis for three consecutive days, resulting in neurologic improvement. Additional competing causes of altered mental status and encephalopathy were investigated and ruled out over the course of a five-day hospitalization. The patient was subsequently discharged with a diagnosis of VAN. This case underscores the importance of proper renal dose adjustment and medication safety in patients with ESRD to prevent serious, avoidable adverse drug events.

## 1. Introduction

Valacyclovir is an antiviral prodrug of acyclovir, commonly prescribed for the treatment and prophylaxis of herpes simplex virus (HSV) and varicella-zoster virus (VZV) infections, including shingles [1]. After oral administration, valacyclovir is rapidly converted to acyclovir, which inhibits viral deoxyribonucleic acid (DNA) polymerase, preventing viral replication. While generally well tolerated in immunocompetent patients, adverse effects can occur, particularly in those with impaired renal function due to accumulation of active metabolites [2]. Valacyclovir-associated neurotoxicity (VAN) is a rare but serious adverse effect characterized by altered mental status, hallucinations, confusion, agitation, and in severe cases, encephalopathy or seizures [3]. These neurotoxic effects are often under-recognized and may be misattributed to primary neurologic or infectious etiologies. The risk of VAN is especially heightened in patients with end-stage renal disease (ESRD), where standard doses can result in toxic plasma levels of acyclovir due to impaired renal clearance [4].

While the standard recommended dosing regimen for valacyclovir in the treatment of herpes zoster infection is 1 g three times daily, valacyclovir requires renal dose adjustments in patients with impaired renal function. For those patients with end stage renal disease (ESRD) and a CrCl < 10 mL/min or those maintained on hemodialysis (HD), the recommended dosing schema is reduced to 500 mg once daily [1]. In this manuscript, we detail a case report wherein a patient with ESRD on HD received a prescription for the standard dosing of valacyclovir, resulting in the development of encephalopathy requiring hospitalization.

## 2. Case Report

Our case describes a 69-year-old Hispanic female with a past medical history of hypertension, type 2 diabetes mellitus, coronary artery bypass grafting performed four years prior to admission, coronary stent placement two years prior to admission, transcatheter aortic valve replacement performed two years prior to admission, and ESRD on HD. Of note, the patient had tested positive for VZV IgG in the past and had completed their zoster vaccination series three years prior to this index hospitalization. At baseline, the patient was cognitively intact and able to manage all activities of daily living, including dressing, cooking, and bathing.

Two days prior to hospitalization, the patient presented to an external outpatient clinic outside of our health system with a chief complaint of a rash on her left foot. At that clinic visit, the patient was diagnosed with herpes zoster reactivation and cellulitis and was issued prescriptions for valacyclovir 1 g three times a day and cephalexin 500 mg three times a day. Outpatient pharmacy records indicate these prescriptions were dispensed to the patient the same day as this clinic visit.

The patient subsequently presented to our emergency department early in the morning of hospitalization day #1, accompanied by their next of kin for new-onset confusion, dizziness, slurred speech, and unusual sensations described by the patient as “brain itching” and like being in “someone else’s skin.” The patient’s physical examination demonstrated waxing and waning mentation with an intermittent ability to appropriately respond to basic questioning. Examination of her lower extremities revealed a bilateral violaceous maculopapular rash on the heels and soles of the feet with no vesicles or excoriation. Additional objective assessments at this point included stable vital signs, serum glucose of 406 mg/dL, potassium of 5.6 mEq/L, sodium of 131 mEq/L (corrected to 138 mEq/L for concomitant hyperglycemia), a blood urea nitrogen of 41 mg/dL, and a white blood cell count of 8.6 × 10^3^ cells/mm^3^. Serum alcohol levels were undetectable, and the urine drug screen was negative. Computed tomography of the head demonstrated no acute intracranial abnormality. Given the patient’s renal impairment, altered mental status, initial uncertainty by the inpatient treatment team of the patient’s home cephalexin dose, and known recent exposure to a dose-unadjusted course of valacyclovir, the patient was treated with a presumptive diagnosis of VAN. Valacyclovir and cephalexin were discontinued, and the patient was admitted to an internal medicine service team with nephrology specialists consulted. A daily account of the patient’s symptoms and laboratory values may be found in Table 1.

The patient was treated with subcutaneous insulin and hemodialysis on hospitalization day #1, with improvement of the above-noted hyperglycemia to 169 mg/dL and hyperkalemia to 4.8 mEq/L three hours later. Interval improvement in the patient’s confusion was noted on hospitalization day #2. The patient’s chronic home medications of aspirin, atorvastatin, carvedilol, gabapentin, hydralazine, and nifedipine were all reinitiated at that time, and daily hemodialysis was continued through hospitalization day #3. By hospitalization day #4, the patient had displayed marked symptomatic improvement, with family and staff noting a return to near-baseline cognition. Magnetic resonance imaging of the brain obtained this day revealed a historical infarct but no acute infarct or encephalitis.

On hospitalization day #5, the patient’s cognition had completely resolved to baseline with orientation to person, place, time, and situation. At that time, nephrology consultants deemed the patient suitable to re-initiate her usual three times per week dialysis schedule. The patient was discharged that day with a diagnosis of encephalopathy attributed to valacyclovir toxicity in the setting of ESRD and inappropriately high medication dosing. The patient was discharged to a skilled nursing facility and, at the time of writing this report, experienced no recurrence of encephalopathic symptoms. The patient was not treated with any additional antivirals. Subsequent medical encounters after hospital discharge have refined the diagnostic hypothesis as to the patient’s initial lower extremity rash and skin discoloration to be secondary to peripheral vascular disease.

## 3. Discussion

There are several important learning points from this case, the first of which is that valacyclovir-induced neurotoxicity (VAN) is a rare but potentially reversible cause of acute encephalopathy, especially in patients with end-stage renal disease (ESRD) receiving hemodialysis. Additionally, we propose that the presence of dissociative symptoms should be investigated in those patients with suspected VAN. Lastly, this case underscores the importance of appropriate diagnostic stewardship in the approach to patients with herpes zoster reactivation.

Valacyclovir is routinely used in the treatment of herpes zoster reactivation and is generally considered to have a wide therapeutic index. Common adverse reactions with valacyclovir include headache and gastrointestinal distress. Rare but severe adverse reactions associated with valacyclovir include acute kidney injury and VAN. The proposed pathophysiologic mechanism of VAN is through the accumulation of the neurotoxic metabolite, 9-carboxymethoxymethylguanine (CMMG) in the serum and cerebrospinal fluid [2,5]. While the biochemical basis of CMMG’s neurotoxicity is incompletely understood, it is believed to inhibit mitochondrial DNA polymerase [4]. Clinical manifestations of VAN include altered level of consciousness, dysarthria, and hallucinations, usually occurring within 72 h of medication initiation [6]. One review of a series of 20 VAN cases additionally noted that “death delusion,” the finding that a patient was convinced they were either going to die or were already dead, occurred frequently with VAN and has been proposed to be characteristic of VAN [6].

Treatment of VAN consists of supportive care and discontinuation of the offending antiviral [3]. Hemodialysis has been reported to reduce serum acyclovir concentrations between 33 and 50% and may be considered for the treatment of VAN in patients with impaired kidney function and established HD access [1,3,7]. In most instances of VAN, complete resolution of symptoms occurs within 7 days of recognition and treatment [3]. This hallmark constellation of VAN symptomologies closely mirrors what we observed in our case. The time to symptom onset in our case, that is to say, the difference in time between when valacyclovir was prescribed and when the patient’s next of kin sought medical services, was two days. Once identified and treatment was initiated, the patient had a complete symptomatic response within five days. These timeframes are consistent with the above-noted time courses of VAN onset and resolution. Furthermore, the patient’s symptoms of confusion, slurred speech, and “brain itching” are consistent with the VAN symptoms of altered level of consciousness, dysarthria, and tactile hallucinations.

Many factors of the patient’s presentation are similar to previously documented instances of VAN. However, the patient’s simultaneous prescription of a standard-dose course of cephalexin serves as a diagnostic confounder in this case. The cephalosporin antibiotic class has also been described to have neurotoxic effects that may lead to encephalopathy [8]. While the majority of data addresses cefepime as a cause of neurotoxicity, case reports of cephalexin-associated delirium have been reported [9,10]. Due to the presence of previous reports of VAN (+1), development of symptoms after valacyclovir initiation (+2), symptoms documented in an objective physical exam (+1), symptomatic improvement following valacyclovir discontinuation (+1), and the presence of other possible causes for the patient’s confusion (−1), we calculated a Naranjo Adverse Drug Reaction Probability Scale score of 4, indicating this to be a possible adverse drug reaction [11].

Our case underscores the importance of quality pharmacovigilance in patients maintained on HD. Appropriate medication dosing in patients with renal dysfunction is within the scope of practice of both physicians and pharmacists [12]. While outpatient providers may infrequently have serum creatinine laboratory data available at the point of care to determine exact dosing schema, patients maintained on hemodialysis may be identified by a cursory history and physical examination and generally prompt specific medication dosing regimens. For valacyclovir, the recommended dose for such a patient is 500 mg daily given after dialysis on days when HD is conducted [1]. The primary risk factors for VAN are higher doses of valacyclovir, increasing age, and declining renal function [3,6,13]. Our patient’s specific factors of advanced age, ESRD on HD, and the receipt of a 1 g three times daily course of therapy each served as risk factors for the development of VAN. Valacyclovir overdose has been reported commonly in patients with ESRD, with one report illustrating that patients on HD received dose-unadjusted prescriptions of valacyclovir in 60% of assessed treatment courses [13]. To overcome this, systematic adoption of dose adjustment strategies for patients with ESRD maintained on HD by both physicians and pharmacists may help mitigate the risk of VAN. Clinical models wherein pharmacists are granted protocolized authority to dose medications in inpatient and outpatient settings have previously demonstrated increased adherence to appropriate medication dosing practices [14,15].

While the hallmark symptoms of VAN are the above-described findings of dysarthria, confusion, and hallucinations, our patient described an out-of-body experience. A minority of previous reports have addressed the finding of dissociative symptoms in patients with VAN. In one of these cases, a 72-year-old man maintained on HD received valacyclovir 1 g three times a day and subsequently presented to inpatient services for VAN [16]. While the patient’s verbiage was not detailed in their own words, the patient was reported to have experienced dissociative symptoms. Additionally, a 24-year-old female on peritoneal dialysis presented with VAN after taking valacyclovir 1 g twice daily for 3 days [17]. During the initial presentation to medical services, the patient stated they were experiencing a sensation of being “outside her own body.” To the best of our knowledge, our report is the third to identify the subjective symptom of bodily dissociation as a component of VAN. Clinical investigation into this specific symptom may refine diagnostic hypotheses to VAN specifically in the correct clinical context.

Our patient’s initial presentation to an external health facility with subsequent diagnosis and treatment for presumed herpes zoster reactivation was subsequently questioned by the inpatient care team, highlighting the importance of diagnostic stewardship in the approach to patients with suspected herpes zoster reactivation. Herpes zoster classically presents as a unilateral painful vesicular rash with a clear dermatomal distribution [18]. Upon presentation to our hospital, the patient’s lower extremities revealed a bilateral violaceous rash without a specific dermatomal distribution. While the manner and extent of the patient’s rash progression from their outpatient diagnosis of herpes zoster reactivation to inpatient presentation for altered mental status cannot be exactly quantified, vascular insufficiency, as may be seen in older adults with cardiovascular disease, may have obfuscated the clinical diagnosis of herpes zoster reactivation. Atypical rashes suspected of being herpes zoster may be tested by polymerase chain reaction to confirm the diagnosis prior to treatment [19].

## 4. Conclusions

It is possible that the patient’s acute altered mental status was the result of VAN. Clinicians should exercise caution and cognizance of VAN risk factors when prescribing valacyclovir. Recent history of valacyclovir use should be considered in the diagnostic approach of patients with an acute onset of confusion and encephalopathy.

## Figures and Tables

**Table 1:** Day-by-day clinical developments.

Day	Symptoms	Laboratory Values	Treatment and/or Imaging
Two days prior to hospitalization	Left heel pain Left foot discoloration Left lower leg papular rash	None obtained	Valacyclovir 1000 mg three times daily initiated Cephalexin 500 mg three times daily initiated
One day prior to hospitalization	No interaction with the healthcare system	N/A	N/A
Hospitalization day #1	Disassociation Disorientation Dizziness Itchiness	Sodium 131 Potassium 5.6 BUN 41 Glucose 406 mg/dL WBC 8.6 × 10^3^ TSH 2.312 mIUmL	CT Head with no intracranial abnormalities Valacyclovir and cephalexin discontinued Hemodialysis conducted
Hospitalization day #2	Confusion Dissociation Disorientation Dizziness	Sodium 135 Potassium 4.0 BUN 10 Glucose 93 mg/dL WBC 8.2 × 10^3^	Hemodialysis conducted
Hospitalization day #3	Oriented to self and location	Sodium 135 Potassium 4.1 BUN 9 Glucose 68 mg/dL WBC 6.2 × 10^3^	Hemodialysis conducted
Hospitalization day #4	Oriented to self, location, time, and event Dizziness	Sodium 132 Potassium 4.4 BUN 10 Glucose 174 mg/dL WBC 6.8 × 10^3^	MRI brain with evidence of previous infarction without acute infarction or encephalitis
Hospitalization day #5	Oriented to self, location, time, and event	Sodium 136 Potassium 4.4 BUN 24 Glucose 102 mg/dL WBC 7.6 × 10^3^	Discharged from the hospital

Abbreviation: N/A, not applicable.

## Data Availability

The original contributions presented in this study are included in the article. Further inquiries can be directed to the corresponding author.
